# Predictive Value of the Risk Assessment and Prediction Tool (RAPT) Score for Primary Hip and Knee Arthroplasty Patients: A Single-Center Study

**DOI:** 10.7759/cureus.14112

**Published:** 2021-03-25

**Authors:** Awf A Alshahwani, Maurice Dungey, Christopher Lillie, Steve Krikler, Christos Plakogiannis

**Affiliations:** 1 Trauma and Orthopaedics, Leicester University Hospital, Leicester, GBR; 2 Trauma and Orthopaedics, Kettering General Hospital, Kettering, GBR; 3 Surgery, Kettering General Hospital, Kettering, GBR

**Keywords:** rapt, eras, predictive, arthroplasty

## Abstract

The Risk Assessment and Prediction Tool (RAPT) was developed to predict patient discharge destination for arthroplasty operations. However, since Enhanced Recovery After Surgery (ERAS) programs have been utilized in the UK, the RAPT score has not been validated for use. The aim of the current study was to evaluate the predictive validity of the RAPT score in an ERAS environment with short length of stay. Data were compiled from 545 patients receiving a primary elective total hip or total knee arthroplasty in a district general hospital over 12 months. RAPT scores, length of stay, and discharge destinations were recorded.

Patients were classified as low, intermediate, or high risk as per their RAPT score. Length of stay was significantly different between groups (p = 0.008), with low-risk patients having shorter length of stay. However, RAPT scores did not predict discharge destination; the overall correct prediction was only 31.9%. Furthermore, the most likely discharge destination was directly home in ≤3 days in all groups (68.5%, 60.2%, and 40% for the low-, intermediate-, and high-risk groups, respectively).

The RAPT score is not an adequate tool to predict the discharge disposition following primary total knee and hip replacement surgery in a UK hospital with a standardized modern ERAS program. Alternative predictive tools are required.

## Introduction

Hip and knee arthroplasties are among the most commonly performed orthopedic operations, and their number increases every year in the UK [1]. The introduction of Enhanced Recovery After Surgery (ERAS) programs over the past decade and standardization of patients’ management have led to improvements in patient-reported outcomes and reduction in the length of stay (LOS) [2].

The increase in the demand for hip and knee arthroplasty, the drive for quick turnaround, and improved utilization of resources are putting healthcare systems under significant pressure. It would be helpful to have tools that can predict LOS and discharge disposition as this would help with allocation of resources and pre-emptively address problems with patients’ discharge, especially with the increasing numbers of joint replacement surgeries every year [1].

The Risk Assessment and Prediction Tool (RAPT) score is a prediction tool that was developed by Oldmeadow et al. [3] in 2003. Since its publication, the score has been validated in several countries. It has been utilized in Europe by Dauty et al. and Coudeyre et al., in which both revealed strong predictive value of the score regarding the LOS and the discharge destination [4,5]. The reports from the US were contradicting, where Dibra et al. [6] and Hansen et al. [7] found the score reliable for discharge planning and to identify the need for extended postoperative rehabilitation for low score patients. On the other hand, in the US as well, Cizmic et al. [8] revealed low predictive accuracy for discharge destination for patients with extended LOS despite good prediction for the LOS itself. This score was also reliable in Asia, where Tan et al. found the score reliable for knee arthroplasty patients to determine the LOS and the discharge destination [9].

In the UK, the score has been found to be predictive for discharge predisposition for lumbar fusion patients, although it has not been validated on a wide base for joint arthroplasty; it's use has been important in the early design of ERAS programs in the UK [2,10].

The score comprises a 12-point scoring system constructed to predict discharge disposition after total hip and knee arthroplasty (THA/TKA). The score was developed and tested on an Australian cohort of 650 patients. By means of an independent t-test, the original authors identified six factors correlated to discharge disposition [3].

The RAPT score has been utilized in our unit since our ERAS pathway for lower limb arthroplasty was redesigned in 2018, and the results have been prospectively collected.

The aim of the current study was to evaluate the predictive validity of the RAPT score in an ERAS environment with short LOS. To our knowledge, there have been no such previous studies in the UK.

## Materials and methods

This retrospective review was approved by the local hospital audit department. Data were prospectively collected over a 12-month period, from September 1, 2018, to August 31, 2019, for all patients undergoing a primary THA/TKA in a district general hospital.

RAPT scores were calculated when patients came to their preoperative educational session (joint school). Patients were categorized as low risk (score: 10-12), medium risk (score: 6-9), or high risk (score: 1-5) according to their score (Table 1). Post-operative LOS and discharge destination were recorded. Discharge destination was categorized as either discharge straight home (LOS equal to or less than median: ≤3 days), additional intervention to discharge home, or admission for prolonged rehabilitation (LOS greater than median: >3 days). The additional interventions available to this ERAS program included an NHS (National Health Service) community team and a contracted community team, both consisting of nurses and therapists. Patients with incomplete data were excluded from further analysis.

**Table 1:** Risk Assessment and Predictive Tool for knee arthroplasty (2003). Score < 6: high-risk prediction (discharge extended to inpatient rehabilitation). Score > 9: low-risk prediction (discharge directly home). Score 6–9: medium-risk prediction.

	Value	score
What is your age group?	50–65 years	2
	66–75 years	1
	>76 years	0
Gender?	Male	2
	Female	1
How far, on average, can you walk? (a block is 200 meters)	Two blocks or more ( rests)	2
	1–2 blocks (the shopping center)	1
	Housebound (most of the time)	0
Which gait aid do you use? (more often than not)	None	2
	Single-point stick	1
	Crutches/frame	0
Do you use community supports? (home help, meals-on-wheels, district nurse)	None or one per week	1
	Two or more per week	0
Will you live with someone who can care for you after the operation	Yes	3
	No	0
Your score (out of 12)		

Statistical analysis was performed using the Statistical Package for Social Sciences (SPSS) Version 25 (IBM Corp., Armonk, NY, USA) [11]. Normality was assessed using the Shapiro-Wilk test [12]. All scale data were non-normally distributed and was analyzed using non-parametric tests. Therefore, data are presented as median (interquartile range) and number (percentage) as appropriate. Differences between groups (as per RAPT score) were assessed using the Kruskal-Wallis one-way analysis of variance. Chi squares (χ2) were used to assess differences between categorical data. Where a significant difference was found, post hoc analysis was performed to identify specific significant differences, which were adjusted for multiple comparisons using the Bonferroni method. Correlations between scale variables were assessed using Spearman’s rho. Multivariate linear regression was used to assess the predictors of LOS. A p-value of <0.05 was accepted as statistically significant.

## Results

**Figure 1:**
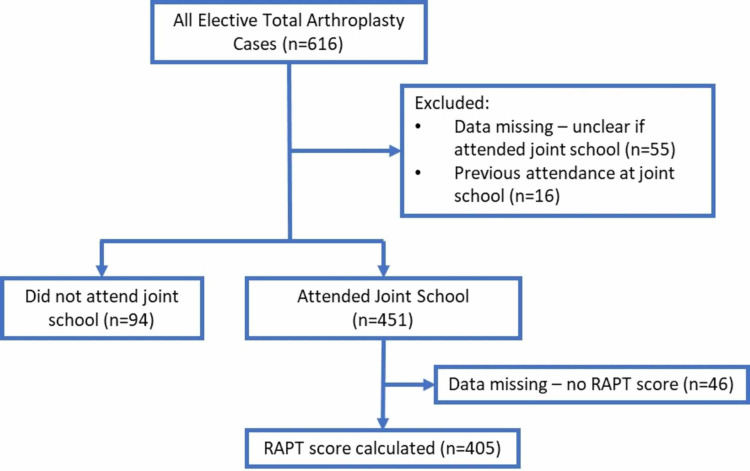
Flow diagram RAPT, Risk Assessment and Prediction Tool

All primary TKA or THA patients were invited to a joint school prior to their operation. A total of 616 patients were booked for TKA or THA. After excluding patients who did not attend the joint school or for whom the records were incomplete, 545 patients remained, of which 405 patients attended the joint school and had a documented RAPT score. This is detailed in Figure 1, and patients’ characteristics and attendance are shown in Table 2.

**Table 2:** Patient characteristics Data are presented as median (interquartile range) and as number (percentage) as appropriate. TKA, total knee arthroplasty; THR, total hip replacement

Patient characteristics (n=405)	
Age (years)	71.0 (63.0-76.5)
Gender	
Male	175 (43.2%)
Female	230 (56.8%)
Operation	
TKA	240 (59.3%)
THR	165 (40.7%)

Patients were categorized as low risk, medium risk, and high risk as per their RAPT score (Table 3). There were significant differences in age and sex between groups. The low-risk group had a greater proportion of male patients than the high-risk group, and the average age of the low-risk group was younger. These differences were expected as these variables feature in the RAPT score.

**Table 3:** Low-, medium-, and high-risk patients as characterised by pre-operative RAPT score. Data are presented as median (interquartile range) or as number (percentage). RAPT, Risk Assessment and Prediction Tool; H, Kruskal-Wallis H test; TKA, total knee arthroplasty; THR, total hip replacement

	Low risk (RAPT: 10-12), n = 149	Medium risk (RAPT: 6-9), n = 226	High risk (RAPT: 1-5), n = 30	p-Value
RAPT score	10.0 (10.0-11.0)	8.00 (7.00-9.00)	4.00 (3.00-5.00)	H = 322.7; p < 0.001
Age (years)	65.0 (60.0-72.0)	73 (67.0-78.0)	76.0 (68.8-81.0)	H = 49.76; p < 0.001
Sex				
Male	90 (60.4%)	82 (36.3%)	3 (10%)	χ^2^= 35.9; p < 0.001
Female	59 (39.6%)	144 (63.7%)	27 (90%)	
Operation				
TKA	88 (59.1%)	135 (59.7%)	17 (56.7%)	χ^2^= 0.12; p = 0.943
THR	61 (40.9%)	91 (40.3%)	13 (43.3%)	
Length of stay (days)	3.00 (2.00-4.00)	3.00 (3.00-4.00)	4.00 (3.00-6.00)	H = 12.29; p = 0.002
Discharge				
Directly home (≤3 days)	102 (68.5%)	136 (60.2%)	12 (40%)	χ^2^= 24.02; p < 0.001
Additional intervention	4 (2.68%)	17 (7.52%)	(26.7%) 10	
Prolonged rehab (>3 days)	43 (28.9%)	73 (32.3%)	8 (33.3%)	
Correct discharge prediction	68.5% directly home (≤3 days)	7.52% additional intervention	33.3.% prolonged rehab (>3 days)	χ^2^= 154; p < 0.001

Length of stay

There was a significant difference in the LOS between groups (H = 12.3; p = 0.002). Post hoc analysis with Bonferroni correction revealed these differences were maintained when comparing low- and medium-risk groups (Z = 2.64; p = 0.024) and low- and high-risk groups (Z = 2.94; p = 0.009), but not between medium- and high-risk groups (Z = 1.80; p = 0.21).

Discharge destination

Chi squares showed a difference in discharge destination between groups (χ^2^ = 24.02; p < 0.001); post hoc analysis found that the proportion of patients requiring additional intervention on discharge in the high-risk group (26.7%) was significantly different from the medium and low-risk groups (p < 0.001). However, no other significant differences in discharge destination were found (p > 0.05).

Patients in the low-risk group had a greater correct discharge prediction (68.5%) than those in the medium-risk (7.52%) and high-risk groups (33.3%) (χ^2^ = 154; p < 0.001); this suggests that the RAPT score is more effective at identifying needs in low-risk patients. It is particularly poor at identifying those in need of additional intervention on discharge.

Discharge destination was not different between males and females (χ^2^ = 0.368; p = 0.832) nor different between THA and TKA (χ^2^ = 4.055; p = 0.132).

## Discussion

These results suggest that the RAPT score was predictive of the LOS but not the discharge destination in a District General Hospital in the UK with an ERAS program and has a median LOS of 3 days; a lower RAPT score was related to longer LOS. However, the predictive categories of the RAPT score were not found to be accurate. In our cohort, the overall correct prediction was only 31.9%.

The most likely discharge destination was directly home in ≤3 days in all groups (68.5%, 60.2%, and 40% for the low-, medium-, and high-risk groups, respectively).

The possibility for requirement of prolonged inpatient rehabilitation was similar in all groups (28.9%, 32.3%, and 33.3% for the low-, medium-, and high-risk groups, respectively).

More than a quarter (26.7%) of the patients in the high-risk group required additional intervention for discharge directly home, whereas only 7.52% of the patients in the medium-risk group were in need of such intervention; this prediction failure had the highest impact in resource planning for our service.

Reviewing previous literature revealed slight variation of assessment, as some institute validated the score individually on knee arthroplasty, such as Dauty et al [4], who reviewed 272 patients and those who had a score of less than 6 were most likely to need a rehabilitation center postoperatively. Similarly, Tan et al [9]. reviewed 569 patients in multiple centers and also the score was predictive regarding LOS and discharge destination but with low predictability with high-risk group. Both of these studies showed longer LOS as they were conducted before the era of ERAS. On the other hand, in the study by Coudeyre et al. [5]., who looked only into the cohort of hip arthroplasty patients, the RAPT sore was found to be predictive in terms of discharge destination and LOS, but it is worthwhile to mention that the LOS was 10 ± 3 days and the patient preference was added regarding the discharge destination, which could impact the results. Therefore, the aforementioned studies differ from our study in that our study revealed that median LOS was much less reflecting the positive effect of ERAS and thereby the negative impact of the predictability of the score.

As the structure and the funding of the healthcare system in the US is fundamentally different from the one in the UK, this would probably explain the difference in the predictive value of RAPT and even difference among multiple institutes in the US. Hansen et al. after reviewing 3,213 patients after THA and TKA revealed a strong prediction of RAPT to pick up those who can directly go home. Similar results were reported by Dibra et al., who validated the predictive value of the score in terms of discharge destination; however, they recommended modifying the score as the predictive expectation increased the predictive value [6,7] . On the contrary , and in the US as well Cizmic et al look mainly into the patient with extended stay over 3 days and found that the score is less predictive regarding discharge destination in those cohort , which probably coincide though partially with our study [8]. Sisac et al. highlighted the positive effect of preoperative education that is targeted at high-risk patients in which the LOS was reduced, which affects the predictive value of the score, by shortening LOS in high-risk individual; this has been demonstrated in our study as well, as the score was already recorded preoperatively in the educational sessions before the procedure , i.e., the joint school [13].

In a recent systematic review, Sconza et al. assessed the validity of the score in multiple institutes. They noted that there were differences in criteria used to discharge the patient, the role of insurance coverage (which in turn depends on the health care system in each country), and, finally, the rehabilitation care the patient receives in the post-acute settings, all of which affect the LOS. As a result, they emphasized the need to validate the score in different countries [14].

The studies that have previously shown the validity of the RAPT score were performed early in the introduction of ERAS programs for arthroplasty (as illustrated by the relatively high LOS in these cohorts). Oldmeadow et al. and Sisak et al. have previously shown that ERAS intervention can change the outcome from the RAPT prediction [3,13].

A very recent study by Cohen et al. revealed that the accuracy of predicting discharge destination would be improved by modifying the cutoff point of the risk group [15].

Our study shows that the intervention and impact of modern ERAS programs on patients’ outcomes make the RAPT score non-predictive. Machine learning algorithms for the prediction of LOS and discharge destination are a promising development; currently, their use is not widely available [16].

## Conclusions

Our study demonstrates that contemporary ERAS intervention and reduction of LOS to a median of less than four days reduces the predictive value of the RAPT score.

We believe that for the NHS in the UK, the RAPT score is not an adequate tool to predict the discharge disposition following primary total knee and hip replacement surgery. We have now stopped using the RAPT and have started exploring alternative prediction tools.
